# Automated Category and Trend Analysis of Scientific Articles on Ophthalmology Using Large Language Models: Development and Usability Study

**DOI:** 10.2196/52462

**Published:** 2024-03-22

**Authors:** Hina Raja, Asim Munawar, Nikolaos Mylonas, Mohammad Delsoz, Yeganeh Madadi, Muhammad Elahi, Amr Hassan, Hashem Abu Serhan, Onur Inam, Luis Hernandez, Hao Chen, Sang Tran, Wuqaas Munir, Alaa Abd-Alrazaq, Siamak Yousefi

**Affiliations:** 1 Department of Ophthalmology, University of Tennessee Health Science Center Memphis, TN United States; 2 Watson Research Center, IBM Research New York, NY United States; 3 School of Informatics, Aristotle University of Thessaloniki Thessaloniki Greece; 4 Quillen College of Medicine, East Tennessee State University Johnson, TN United States; 5 Gavin Herbert Eye Institute, School of Medicine, University of California Irvine, CA United States; 6 Department of Ophthalmology, Hamad Medical Corporation Doha Qatar; 7 Edward S. Harkness Eye Institute, Vagelos College of Physicians and Surgeons, Columbia University Irving Medical Center New York, NY United States; 8 Department of Biophysics, Faculty of Medicine, Gazi University Ankara Turkey; 9 Association to Prevent Blindness in Mexico Ciudad Mexico; 10 Department of Pharmacology, Addiction Science and Toxicology, University of Tennessee Health Science Center Memphis, TN United States; 11 Department of Ophthalmology and Visual Sciences, School of Medicine, University of Maryland Baltimore, MD United States; 12 AI Center for Precision Health, Weill Cornell Medicine-Qatar Doha Qatar

**Keywords:** Bidirectional and Auto-Regressive Transformers, BART, bidirectional encoder representations from transformers, BERT, ophthalmology, text classification, large language model, LLM, trend analysis

## Abstract

**Background:**

In this paper, we present an automated method for article classification, leveraging the power of large language models (LLMs).

**Objective:**

The aim of this study is to evaluate the applicability of various LLMs based on textual content of scientific ophthalmology papers.

**Methods:**

We developed a model based on natural language processing techniques, including advanced LLMs, to process and analyze the textual content of scientific papers. Specifically, we used zero-shot learning LLMs and compared Bidirectional and Auto-Regressive Transformers (BART) and its variants with Bidirectional Encoder Representations from Transformers (BERT) and its variants, such as distilBERT, SciBERT, PubmedBERT, and BioBERT. To evaluate the LLMs, we compiled a data set (retinal diseases [RenD] ) of 1000 ocular disease–related articles, which were expertly annotated by a panel of 6 specialists into 19 distinct categories. In addition to the classification of articles, we also performed analysis on different classified groups to find the patterns and trends in the field.

**Results:**

The classification results demonstrate the effectiveness of LLMs in categorizing a large number of ophthalmology papers without human intervention. The model achieved a mean accuracy of 0.86 and a mean *F*_1_-score of 0.85 based on the RenD data set.

**Conclusions:**

The proposed framework achieves notable improvements in both accuracy and efficiency. Its application in the domain of ophthalmology showcases its potential for knowledge organization and retrieval. We performed a trend analysis that enables researchers and clinicians to easily categorize and retrieve relevant papers, saving time and effort in literature review and information gathering as well as identification of emerging scientific trends within different disciplines. Moreover, the extendibility of the model to other scientific fields broadens its impact in facilitating research and trend analysis across diverse disciplines.

## Introduction

### Background

A literature review is an integral component of the research process that involves systematically reviewing, evaluating, and synthesizing existing scholarly publications from databases such as MEDLINE (PubMed), Embase, and Google Scholar. The standard approach for a literature review involves using a bibliographic search engine to conduct an initial comprehensive search. Researchers use relevant keywords and filters, including clinical query filters, to retrieve a wide range of articles. The next steps include manually screening the retrieved articles by reviewing titles; abstracts; and, in most cases, full texts to assess their relevance and inclusion criteria. This combination of automated search and manual screening ensures a thorough review while targeting specific research objectives.

However, a literature review can be a challenging and time-consuming task for researchers, requiring meticulous examination of numerous sources and a critical analysis of their findings. The process demands substantial time and effort to effectively navigate through the vast expanse of scholarly literature from different databases and extract meaningful insights. Artificial intelligence tools have been used to facilitate this search process [1].

### Classical Methods

Machine learning models have been applied to perform the text classification task based on feature engineering [2-4]. A semiautomated model was proposed for article classification in systemic review articles based on mechanistic pathways [5]. A total of 24,737 abstracts from both the PubMed and Web of Science databases and 861 references were found to be relevant. They evaluated the Naïve Bayes, support vector machines, regularized logistic regressions, neural networks, random forest, LogitBoost, and XGBoost models. The best-performing model achieved a sensitivity and specificity of approximately 70% and approximately 60%, respectively. Kanegasaki et al [6] used long short-term memory networks for the classification of abstracts. They used 2 data sets with 1307 and 1023 articles and achieved 73% and 77% respectively. These machine learning approaches were primarily based on feature engineering, which requires domain expertise.

### Supervised Natural Language Processing Models

Natural language processing (NLP) applications have significantly advanced in recent years and gained tremendous popularity due to their wide range of applications across various domains. With the increasing availability of large data sets and advancements in computational power, NLP has made remarkable progress, revolutionizing the way we interact with technology [7-10]. In particular, NLP has gained interest in the field of information retrieval. NLP techniques, such as keyword extraction, document clustering, and semantic search, have improved the accuracy and relevance of the search results.

Hasny et al [11] used the Bidirectional Encoder Representations from Transformers (BERT) model for classifying articles into human, animal, and in vivo groups. Ambalavanan and Devarakonda [12] used SciBERT to classify scientific articles into 4 major categories, including format, human health care, purpose, and rigor. The format category included original studies, reviews, case reports, and general articles. The human health care category encompassed all articles discussing human health. The purpose category included articles discussing etiology, diagnosis, prognosis, treatment, costs, economics, and disease-related prediction. Rigor class included the studies that presented design criteria specific to a class purpose. The model achieved an *F*_1_-score of 0.753 on the publicly available Clinical Hedges data set. Devlin et al [13] used the BERT model for the classification of scientific articles on randomized controlled trials. The BioBERT variant, trained on titles and abstracts, showed the highest performance of 0.90 in terms of the *F*_1_-score. Another study [14] fine-tuned variants of the BERT model, including BERTBASE, BlueBERT, PubMedBERT, and BioBERT, for the classification of human health studies. They used the abstracts and titles of 160,000 articles from the PubMed database. BioBERT showed the best results and achieved a specificity of 60% to 70% and a recall of >90%. The study [15] proposed a weakly supervised classification of biomedical articles. The model was trained on a weakly labeled subset of the biomedical semantic indexing and question answering 2018 data set based on MeSH (Medical Subject Headings) descriptors. BioBERT was used to generate the embedding for words and sentences, and then the cosine similarity was used to assign labels. The proposed model achieved an *F*_1_-score of 0.564 for the BioASQ 2020 data set. BERT and its variant models have shown better performance for the text classification.

### Zero-Shot Learning Methods

Conventional approaches to text classification have traditionally relied on the assumption that there is a fixed set of predefined labels to which a given article can be assigned. However, this assumption is violated when dealing with real-world applications, where the label space for describing a text is unlimited and the potential labels that can be associated with a text span an infinite spectrum, reflecting the diverse and nuanced nature of textual content. Such complexity challenges the conventional methods and calls for innovative strategies to navigate the expansive and unbounded label space. To address these issues, zero-shot techniques [16-18] have been developed and are gaining popularity. Zero-shot learning (ZSL) involves classifying instances into categories without any labeled training data [19]. It leverages auxiliary information such as semantic embeddings or textual descriptions to bridge the gap between known and unknown categories. This enables the models to generalize to novel classes and make predictions for unseen categories. Mylonas et al [20] used zero-shot model for classifying PubMed articles into emerging MeSH descriptors. Instead of using the standard n-grams approach, the method exploited BioBERT embeddings at the sentence level to turn textual input into a new semantic space for the Clinical Hedges data set [21]. Unlike traditional models, the ZSL model does not explicitly require the labeled data; however, these models performed well on downstream tasks.

In this study, we have used large language models (LLMs) that include ZSL for categorizing the ophthalmology articles extracted from the PubMed database into different categories based on title and abstract. We fine-tuned the BERT model and its variants BERTBASE, SciBERT, PubmedBERT, and BioBERT for those categories that did not show good results from the ZSL model. Several powerful models, including Decoding-enhanced BERT with Disentangled Attention (mDeBERTa), Bidirectional and Auto-Regressive Transformers (BART), and the recently introduced Llama 2, present competitive alternatives to ChatGPT. However, Llama 2, despite its potential, imposes significant demands on graphics processing unit and memory resources. Even its smaller variant, with 7 billion parameters, requires substantial computational power, posing challenges for users with limited access to high-performance computing resources. To ensure broader accessibility, we prioritized a model with lower resource requirements. Accordingly, we selected the open-source BART model that is executable on central processing unit, thus providing both acceptable performance and enhanced accessibility to broader users. In addition, we performed a trend analysis based on the classified results, providing researchers with insights into emerging trends in the field to stay updated on the latest developments and identifying key areas of interest. Overall, we provide a method that enhances the efficiency, relevance, and interdisciplinary potential of the literature review process.

The rest of the study is organized as follows: the Methods section presents the materials and methods of the proposed framework for text classification and trend analysis. The Results section discusses the results of the different experiments performed for the evaluation of the proposed model. The Discussion section includes the principal findings and concludes the proposed work.

### Contributions

Various classification models have been proposed in the literature for biomedical articles to retrieve relevant information [1-21]. Fine-tuned BERT and its variants have been used for text classification. Following are the main contributions of the studies:

- We have explored the ZSL models for the classification of biomedical articles.
- We have developed different use cases targeting the field of ophthalmology.
- To evaluate the model, we have generated a data set that includes 1000 articles related to ocular diseases. The articles were manually annotated by 6 experts into 15 categories.
- The ZSL model BART achieved a mean accuracy of 0.86 and an *F*_1_-score of 0.85.
- In addition to the classification of articles, we also performed a trend analysis on different classified groups.
- The model is adaptable to other biomedical disciplines without explicit fine-tuning or training.

## Methods

### Data Set

There are several annotated data sets available for various NLP tasks in the biomedical domain. However, in the field of ophthalmology, there is a scarcity of publicly accessible data sets for performing NLP tasks. To address this gap, we have taken the initiative to curate a data set focused on ocular diseases. Our retinal diseases (RenD) data set comprises 1000 articles sourced from PubMed, covering various conditions such as diabetic retinopathy (DR), glaucoma, diabetic macular edema, age-related macular degeneration, cataract, dry eye, retinal detachment, and central serous retinopathy. To ensure accurate categorization, we enlisted the expertise of 6 domain specialists who meticulously annotated the articles based on abstracts. To ensure accuracy and reliability in the annotation process, each article in our data set is reviewed and annotated by at least 3 individual annotators (refer to Table S1 in Multimedia Appendix 1 for the guidelines for data annotation). This multiple-annotator approach helps mitigate potential biases and inconsistencies that could arise from a single annotator’s perspective. Once the annotation is completed, the final label for each article is determined based on majority voting. Each article was annotated against 28 labels (Table S1 in Multimedia Appendix 1), and due to not having enough samples in some categories, we dropped those in further classification tasks. Thus, we selected 19 categories and grouped them into 4 categories (Table 1).

We will make this data set publicly accessible to the community for advancing research, facilitating comprehensive analysis, enabling more targeted investigations into ocular diseases, and promoting open science.

**Table 1:** Description of the data sets.

Data set and group			Category
**Retinal disease (N_T_^a^=1000 and N_C_^b^=19)**			
	Article type	Clinical, experimental, and automated model	
	Ocular diseases	DR^c^, DME^d^, AMD^e^, glaucoma, dry eye, cataract, CSR^f^, and retinal detachment	
	Clinical studies subclass	Screening, diagnosis, prognosis, etiology, and management	
	Automated studies subclass	Image processing techniques, machine learning models, and deep learning model	
**Dry eye (N_T_=67 and N_C_=6)**			
	Clinical studies subclass	Tear film break up time, infrared thermography, lipid layer interface pattern, meibomian gland study, blink study, tear film assessment, and tear meniscus assessment	
**Glaucoma (DemL; N_T_=115 and N_C_=2)**			
	Automated studies subclass	Machine learning model and deep learning model	

^a^N_T_: the total number of articles in each data set.

^b^N_C_: the total number of categories in each data set.

^c^DR: diabetic retinopathy.

^d^DME: diabetic macular edema.

^e^AMD: age-related macular degeneration.

^f^CSR: central serous retinopathy.

### Study Design

**Figure 1:**
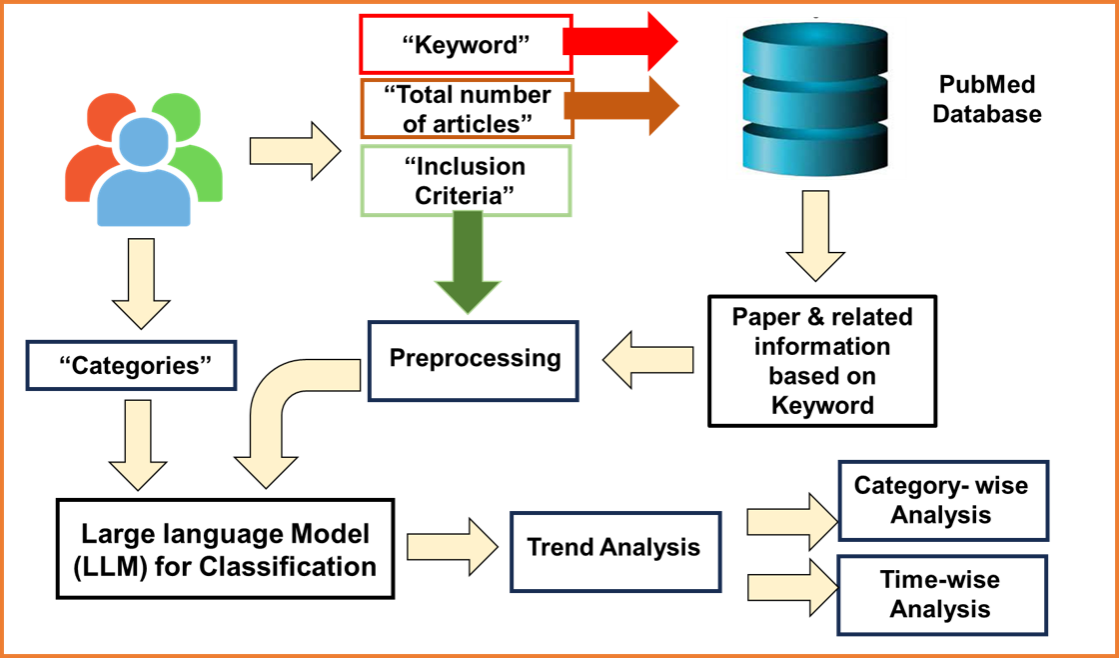
Flow diagram of the proposed framework. The model takes input (keyword, inclusion criteria, and categories for classification), and articles are fetched from PubMed based on keyword. The inclusion criteria are fed into the preprocessing module to select the desired articles from the fetched data. A large language model classifies the articles based on the predefined categories. Finally, trend analysis is performed on classified categories.

Our framework automates the entire literature review process in the ophthalmology domain (Figure 1). More specifically, by incorporating user-defined criteria, including keywords, the number of articles, inclusion criteria, and categories for classification. Our framework performs a systematic retrieval and analysis of relevant articles automatically. Initially, a keyword is fed into PubMed, and the related articles are fetched, including the abstract, title, publication year, and link. The preprocessing step in this context involves the selection of a specific subset of articles from a larger corpus of fetched articles. This selection process is contingent upon the application of predefined inclusion criteria, with a specific temporal constraint. In this study, articles falling within the temporal range spanning from 2015 to 2022 are considered for inclusion in the subsequent analyses. This temporal delimitation serves as a crucial preprocessing measure, narrowing down the data set to a more focused and relevant time frame for the research objectives. The selected articles are then fed into the LLM for classification based on user-defined categories. For LLM, first we targeted using the ZSL models that can classify the text without explicit training. If the ZSL model is unable to achieve better performance, then we fine-tune the BERT model. Finally, after the article classification by LLM, we have performed 2 types of trend analysis.

### Zero-Shot Classification

Zero-shot classification is an approach to predict the class of instances for categories they have never seen during training, using auxiliary information or semantic embeddings. It enables generalization to unseen classes and expands the classification capabilities beyond the limitations of labeled training data. We have used BART [22], which is pretrained based on a sequence-to-sequence model that combines bidirectional and autoregressive techniques for improved text generation and comprehension. BART has 12 transformer layers with a hidden size of 1024 that was initially trained on Wikipedia and the BookCorpus data set and fine-tuned on Multi-Genre Natural Language Inference tasks. BART is an amalgamation of the bidirectional encoder found in BERT and the autoregressive decoder used in Generative Pretrained Transformer (GPT) (details of architecture can be found in Figure S1 in Multimedia Appendix 1). While BERT comprises approximately 110 million trainable parameters and GPT-3 consists of 117 million parameters, BART, being a combination of the 2, has approximately 140 to 400 million parameters. This larger parameter count in BART accommodates its sequenced structure, which incorporates both encoding and decoding capabilities for a wide range of NLP tasks. The model receives the title and abstract of an article as input and generates probabilities for different categories. The final label for multiclass classification of articles is determined by taking the maximum probability among all classes (equation 1), and for multilabel classification, class labels are assigned as probability is greater than the threshold value (equation 2):



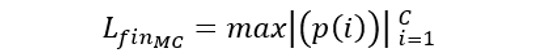

**(1)**




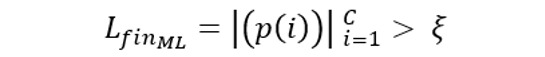

**(2)**


In these equations, *p(i)* is the probability of an article based on the title and abstract, C is the total number of classes for a particular category, and *ξ* is the threshold.

### Fine-Tunning the Classification Model

BERT [13] and its variant models, namely, distilBERT, SciBERT, PubmedBERT, and BioBERT, have been subjected to fine-tuning to address classification tasks for categories in which the ZSL model (BART) is unable to produce more accurate results. The preprocessing stage entails the concatenation of article titles and abstracts, which are subsequently input into the respective BERT model. The model generates probabilities for each class, and if the probability for a specific category is higher than a threshold value, the article is assigned the label corresponding to that category.

### Trend Analysis

In addition to the classification of articles, we performed 2 additional analyses as well. More specifically, we performed a technology trend analysis to obtain valuable insights into the distribution of research across different classes, highlighting classes with higher or lower publication frequencies. This information aids in understanding the emphasis and focus of research efforts, enabling resource allocation, and identifying areas that may require further attention or investigation. We also performed an interest trend analysis to provide a comprehensive view of publication trends over specific periods. By identifying the popularity of techniques or topics over time, this analysis facilitates the detection of emerging trends and the evaluation of long-term patterns. These trend analyses, applicable to all levels of classification categories, contribute to an enhanced understanding of the dynamic nature and evolving landscape of research in the field.

### Ethical Considerations

We have not included any human and animals in our study.

## Results

This section presents the experiments we have performed to evaluate the LLMs for classification.

### Experimental Details

We evaluated our models based on the RenD data set that we annotated. In addition, we evaluated the models to classify categories based on 2 review studies related to dry eye disease and glaucoma (Table 1). We present the results in terms of accuracy, area under the curve (AUC; *F*_1_-score, precision, and recall for each data set. For multilabel classification, we evaluated the model in terms of *F_1_* micro, Pv micro, Re micro, and AUC.

### Ablation Study

#### Overview

Our study encompasses both multiclass and multilabel classification tasks. To accomplish this, we used the ZSL model and fine-tuned the model for which the ZSL was not performing well. Through a series of ablation experiments, we systematically investigated the impact of different settings (refer to the subsequent sections) on the performance of the model. By modifying and assessing various settings, we gained insights into the individual contributions and effects of each setting, allowing us to refine and optimize our approach accordingly.

#### ZSL Model Selection

In our study, we conducted an evaluation of the ZSL state-of-the-art models and multiple variants of the BART model. On the basis of a comprehensive analysis, we identified the model variant that exhibited the most favorable performance in our specific context (Table 2).

We selected the BART model that showed the best performance. In addition, we performed experiments using different keywords for the different categories. It was observed that for the ZSL model, the prompts should be more descriptive and provide some information about related categories to improve the accuracy (Table 3).

For the category “*Clinical, Experimental, and Automated Model*,” we have tested various keywords and found that “*Clinical finding based on humans*,” “*Experimental study based on animals*,” and “*Technical study based on automated model*” keywords showed the best results with an accuracy of 0.91 and an *F*_1_-score of 0.92. During our evaluation, we investigated the potential of using abstracts and titles for classification across various categories. We discovered that classification solely based on titles closely approximates the results obtained from using abstracts for most of the categories. However, the abstract and title together enhance the efficacy of the classification.

**Table 2:** Evaluation of the zero-shot learning classification models for category 1 from the retinal disease (RenD) data set.

	Abstract							Title					
	Time (minutes)	Accuracy	*F*_1_-score	AUC^a^	Precision	Recall	Time (minutes)		Accuracy	*F*_1_-score	AUC	Precision	Recall
BART^b^-base	34.34	0.08	0.03	0.42	0.86	0.08	3.5		0.01	0.005	0.5	0.006	0.01
Bart-large	104.5	0.11	0.03	0.46	0.17	0.115	12.24		0.5	0.57	0.39	0.68	0.50
Bart-large-CNN^c^	37.63	0.08	0.03	0.42	0.86	0.08	4.2		0.36	0.50	0.58	0.84	0.36
Bart-mnli^d^-CNN	231.42	0.74	0.78	0.65	0.84	0.74	17.76		0.09	0.06	0.42	0.2	0.09
mDeBERT^e^a-v3-base	79.28	0.87	0.85	0.74	0.88	0.87	31.06		0.76	0.82	0.80	0.91	0.76
Bart-large-mnli	141.50	0.91	0.92	0.91	0.93	0.91	15.21		0.91	0.82	0.93	0.94	0.91

^a^AUC: area under the curve.

^b^BART: Bidirectional and Auto-Regressive Transformers.

^c^CNN: convolution nerual network

^d^Multi-Genre Natural Language Inference.

^e^BERT: Bidirectional Encoder Representations from Transformers.

**Table 3:** Investigation of prompts for classifying the retinal diseases data set using the Bidirectional and Auto-Regressive Transformers (BART) zero-shot learning model. Articles are explicitly categorized using abstract.

Category and prompt			Abstract											Title								
			Accuracy		*F*_1_-score		AUC^a^		Precision		Recall		Accuracy			*F*_1_-score		AUC		Precision		Recall
**Clinical study, experimental study, and automated model**																						
	“Clinical Study,” “Experimental Study,” and “Automated Studies”	0.80		0.82		0.70		0.85		0.80		0.67			0.76		0.76		0.89		0.67	
	“Clinical Study,” “Experimental Study,” and “Automated Model”	0.80		0.83		0.74		0.86		0.80		0.68			0.77		0.801		0.91		0.68	
	“Clinical Study,” “Experimental Study based on animals,” and “Technical study based on Automated Model”	0.85		0.87		0.91		0.92		0.85		0.85			0.87		0.91		0.92		0.85	
	“*Clinical Finding based on humans,” “Experimental Study based on animals,” and “Technical study based on Automated Model”*^b^	*0.91*		*0.92*		*0.91*		*0.93*		*0.91*		*0.91*			*0.92*		*0.93*		*0.94*		*0.91*	
**Image processing techniques, machine learning models, and deep learning models**																						
	“Deep learning Model,” “Image processing technique,” and “ONLY Machine learning”	0.65		0.54		0.12		0.47		0.65		0.68			0.55		0.05		0.47		0.68	
	“Deep learning Model,” “Image processing technique,” and “Classic Machine learning”	0.66		0,57		0.74		0.79		0.66		0.69			0.60		0.73		0.71		0.69	
	“Deep learning Model,” “Digital Image processing technique,” and “Classic Machine learning”	0.65		0.58		0.68		0.61		0.65		0.66			0.56		0.71		0.76		0.66	
	“*Deep learning Model,” “Digital Image processing technique,” and “Machine learning Model”*	*0.82*		*0.82*		*0.87*		*0.86*		*0.82*		*0.92*			*0.92*		*0.95*		*0.94*		*0.92*	

^a^AUC: area under the curve.

^b^Italicized prompts show the best results for that particular category.

#### Hyperparameters for Fine-Tuning BERT

For the categories in which the ZSL model (BART) provided poor results, we fine-tuned the BERT model and its variants to perform categorization. We conducted hyperparameter tuning based on this to enhance the model’s reliability and significance. By carefully selecting and fine-tuning hyperparameters such as the learning rates, batch sizes, and regularization strengths, we aimed to achieve accurate and meaningful results (Table S2 in Multimedia Appendix 1). For the BioBERT model, we selected a learning rate of 1e-05, a batch size of 8, a maximum length of 400, and a number of epochs of 20.

### Evaluation Results

#### Article Classification Evaluation

This section presents the results based on the metrics that were selected in the ablation experiments. On the basis of the evaluation, BART demonstrated the best performance among the tested models. Therefore, further classification tasks were conducted using the BART model to capitalize on its superior performance. Table 4 shows the classification results using BART for the RenD data set for the categories of article type, ocular diseases, clinical studies subclass, and automated studies subclass.

The article type group was classified into 3 subcategories: clinical, experimental, and automated studies. The BART model demonstrated promising performance for the article type group, with an accuracy of 0.91, an *F*_1_-score of 0.92, an AUC of 0.91, a precision of 0.93, and a recall of 0.91. For the article group type, classification based on only abstract and only title and combination of both are performing consistent. The automated model group is further categorized into image processing techniques, machine learning models, and deep learning models. For the automated study subclass group, the ZSL model achieved the best performance for title-based classification, with accuracy, *F*_1_-score, AUC, precision, and recall of 0.92, 0.92, 0.95, 0.94, and 0.92, respectively. However, the second-best scores were achieved by classification based on abstract and title.

The clinical studies are further categorized into screening, diagnosis, prognosis, etiology, and management, constituting a multilabel classification scenario. However, ZSL achieved the best score of F1 micro of 0.52, AUC of 0.68, precision micro of 0.49, and recall micro of 0.61. The ocular group is classified into DR, diabetic macular edema, age-related macular degeneration, glaucoma, dry eye, cataract, central serous retinopathy, and retinal detachment. In terms of accuracy, *F*_1_-score, AUC, precision, and recall, the classification based on titles yielded the most favorable outcomes. Specifically, the results for the title-based classification were 0.85 accuracy, 0.85 *F*_1_-score, 0.92 AUC, 0.89 precision, and 0.86 recall. Following closely were the results for the abstract-based classification, with values of 0.85 accuracy, 0.83 *F*_1_-score, 0.91 AUC, 0.87 precision, and 0.85 recall.

**Table 4:** Classification of scientific articles from retinal disease (RenD) data set into 4 groups: article type, ocular diseases, clinical studies subclass, and automated studies subclass, which are classified into 3, 8, 4, and 3 categories, respectively.

			Article type (MC^a^)		Ocular diseases (MC)		Clinical studies subclass (ML^b^)		Automated studies subclass (MC)
**Abstract**									
	Accuracy	*0.91* ^c^		0.85^d^		—^e^		0.82	
	*F*_1_-score	*0.92*		0.83^d^		0.49		0.82	
	AUC^f^ (95% CI)	*0.91* (0.89-0.92)		0.91^d^ (0.85-0.92)		0.67 (0.64-0.70)		0.87 (0.85-0.90)	
	Precision	*0.93*		0.87^d^		0.33		0.86	
	Recall	*0.91*		0.85^d^		0.82		0.82	
**Title**									
	Accuracy	*0.91*		*0.85*		—		0.92^d^	
	*F*_1_-score	*0.92*		*0.85*		0.50		0.92^d^	
	AUC (95% CI)	*0.91* (0.87-0.93)		*0.92* (0.86-0.94)		0.67 (0.64-0.71)		0.95^d^ (0.79-0.88)	
	Precision	*0.93*		*0.89*		0.42		0.94^d^	
	Recall	*0.91*		*0.86*		0.61		0.92^d^	
**Probability (abstract+title)**									
	Accuracy	0.85		0.78		—		0.90^e^	
	*F*_1_-score	0.87		0.73		0.51		0.89^e^	
	AUC (95% CI)	0.91 (0.87-0.94)		0.86 (0.79-0.88)		0.67 (0.79-0.88)		0.93^e^ (0.89-0.94)	
	Precision	0.92		0.73		0.42		0.91^e^	
	Recall	0.85		0.78		0.61		0.90^e^	
**Appending title to abstract**									
	Accuracy	0.91^d^		0.84		—		0.90^e^	
	*F*_1_-score	0.91^d^		0.82		0.52		0.89^e^	
	AUC (95% CI)	0.91^d^ (0.86-0.92)		0.90 (0.86-0.92)		0.68 (0.62-0.71)		0.93^e^ (0.90-0.95)	
	Precision	0.93^d^		0.86		0.49		0.91^e^	
	Recall	0.91^d^		0.84		0.61		0.90^e^	

^a^MC: multiclass classification.

^b^ML: multilabel classification.

^c^The best results are italicized.

^d^The second-best scores.

^e^Not available.

^f^AUC: area under the curve.

#### Trend Analysis

**Figure 2:**
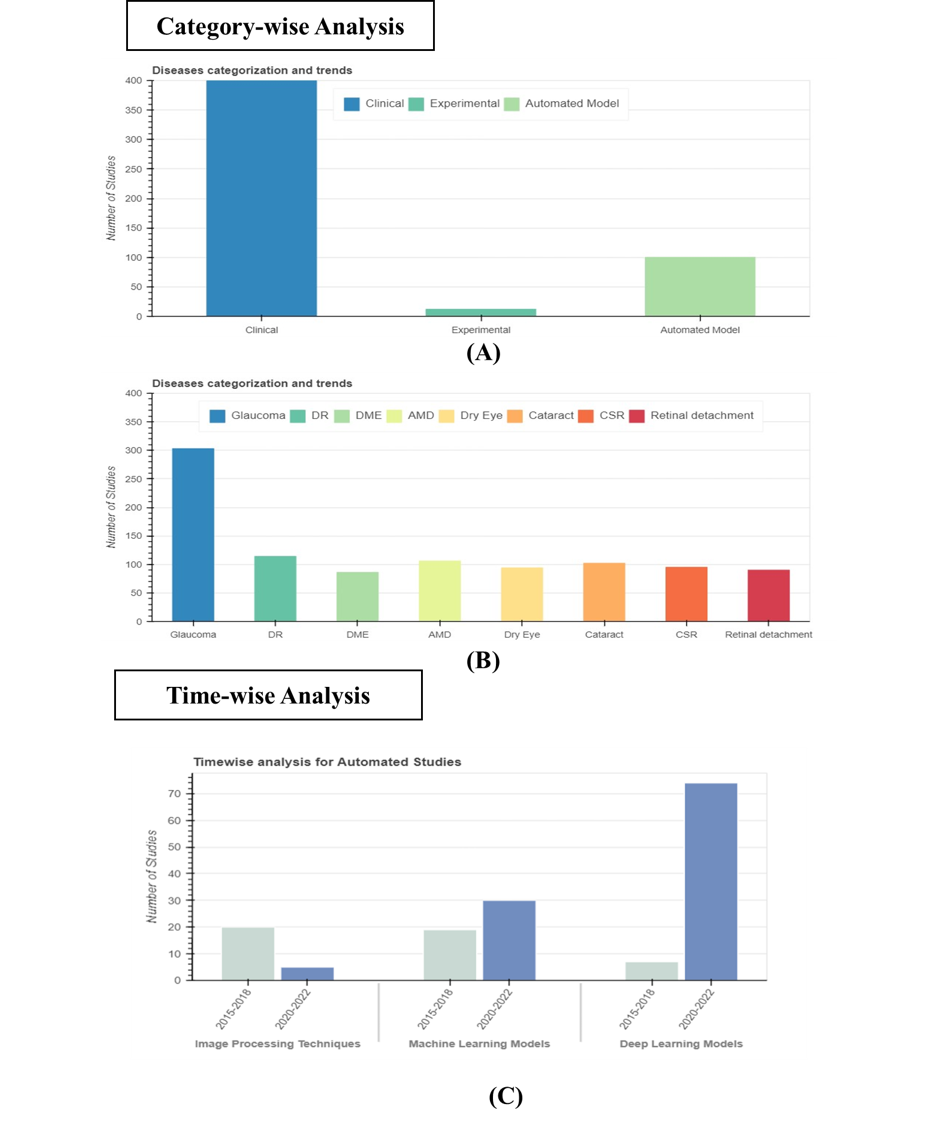
Trend analysis of classified articles: (A) and (B) category-wise analysis for article type and ocular diseases group, respectively, and (C) timewise analysis for automated studies subclass group: image processing techniques, machine, and deep learning models. AMD: age-related macular degeneration; CSR: central serous retinopathy' DME: diabetic macular edema; DR: diabetic retinopathy.

A category-wise analysis was performed for the article type and ocular disease groups based on the RenD data set (Figure 2). Some other results are also reported in Figure S2 in Multimedia Appendix 1.

Category-wise analysis showed more frequent papers on clinical studies compared to experimental- and automated-based studies. For ocular diseases, more studies have discussed DR and glaucoma compared to other ocular diseases. A timewise analysis was conducted for the subgroup of automated studies from 2015 to 2022. The trends indicated that, in the initial years, these studies primarily relied on image processing techniques. However, as time progressed, machine learning gained traction, and eventually, deep learning models became increasingly popular in this field, reflecting how technology is evolving in ophthalmology. For the time-wise analysis of diseases, refer to Multimedia Appendix 1.

## Discussion

### Principal Findings

We have a proposed framework aimed at streamlining the literature review process. This framework entails an automated system that operates by taking user-specified keywords as input. By leveraging these keywords, the system retrieves relevant articles from the PubMed database. In addition, the user specifies the desired categorization for these articles. This approach aims to simplify and expedite the traditionally time-consuming task of conducting literature reviews. We investigated the efficacy of using the LLM model to perform article classification.

LLMs, particularly ChatGPT, have gained huge popularity due to their versatility in performing various tasks, including question answering and trend analysis within specific fields. Moreover, these models demonstrate the capability to generate research papers, letters, and other written content, showcasing their potential for creative text generation. However, the generated content is not always entirely authentic, as it can occasionally produce fake references and links, raising concerns about the reliability and accuracy of the information presented. Therefore, we proposed a framework for automating the literature review process and finding different trends in various disciplines. However, a limitation of ChatGPT-3.5 is the fact that it is not equipped with information beyond September 2021; therefore, it may not provide facts or knowledge beyond this date. We target using open-source LLMs for article classification and then performing category-wise and timewise analysis. Other open-source LLMs have become available recently. For instance, mDeBERTa, BART, and the recently released Llma 2 and its variants may outperform ChatGPT. However, using Llama 2 requires significant graphics processing unit and memory resources. Even the small variant of the model, with 7 billion parameters, demands substantial computational power to function effectively. These resource requirements can pose challenges for users with limited access to high-performance hardware.

Hence, our primary goal is to select a model that not only delivers robust performance but also requires fewer computational resources, thereby enhancing accessibility for a broader user base. The BART model aligns with these criteria as it is open source, allowing seamless execution on central processing unit. This choice ensures a balance between efficiency and performance, making advanced NLP capabilities accessible to a wider community. In a direct comparison with alternative models, BART stands out, showcasing superior overall performance across various evaluation metrics and in terms of computational resources.

### Categorization, Classification, and Trend Analysis

We used BART as the ZSL classifier, and we used the abstract and title separately for article classification. After obtaining the probabilities from each model, we combined the probabilities and performed classification. In addition, we also appended the title to the abstract and added it to the models.

The BART model showed compromising results for the categories article type, ocular diseases, and automated studies subclass of the RenD data set. The classifications based on abstract and title are nearly similar in performance. For clinical studies subclass grouping, the BART achieved an F1 micro score of 0.52 and an AUC of 0.68. To improve the performance for this class, we fine-tuned BERT and its variant, BioBERT, which performed best with an F1 micro score of 0.67 and an AUC of 0.70. The lower performance in this class is likely due to the class imbalance, which typically affects the model’s ability to generalize and accurately predict instances of the minority class.

We also evaluated the performance of the BART model to classify the articles into different categories for 2 undergoing review studies including DEye and DemL. The articles in both the review studies were annotated by reviewing the entire article. However, we just used the abstract and title, and for DEye, our model achieved an AUC of 0.79 and an *F*_1_-score of 0.63. To improve the performance of the BART model, we performed a hierarchical analysis in which the multilabel task is divided into a binary classification. Classification was performed across different thresholds for each class, and the optimal value was chosen based on the best results achieved (Table S3 in Multimedia Appendix 1). The results showed that converting the multilabel problem into binary classification improves the performance of the BART model. In addition to this, we also observed that abstract-based classification and whole article–based annotation provided comparable results for each class (Table 5).

On the basis of the DemL data set, the BART model’s classification is based on the abstract, and the title is as accurate as the whole article–based annotation for the DemL data set.

**Table 5:** Bidirectional and Auto-Regressive Transformers (BART) model evaluation for classification of the Dry eye and DemL data sets.

Data set and category			Classification type		Abstract											Title								
					Accuracy		*F*_1_-score		AUC^a^		Precision		Recall		Accuracy			*F*_1_-score		AUC		Precision		Recall
**Dry eye**																								
	Tear film break up time, infrared thermography, lipid layer interface pattern, meibomian gland study, tear film assessment, and tear meniscus assessment	MC^b^		0.63		0.79		0.60		0.67		0.63		0.44			0.72		0.28		0.84		0.44	
	Tear film break up time	BC^c^		0.91		0.73		0.79		1.0		0.58		0.7			0.47		0.72		0.34		0.75	
	Infrared thermography	BC		0.94^d^		0.77^d^		0.91^d^		0.70^d^		0.89^d^		0.83^d^			0.42^d^		0.69^d^		0.39^d^		0.5^d^	
	Lipid layer interface pattern	BC		0.91		0.54		0.71		0.80		0.44		0.86			0.47		0.68		0.50		0.44	
	Meibomian gland study	BC		0.92		0.87		0.90		0.89		0.85		0.92			0.87		0.90		0.89		0.85	
	Tear film assessment	BC		0.80		0.64		0.80		0.54		0.80		0.71			0.42		0.62		0.38		0.46	
	Tear meniscus assessment	BC		*0.98* ^e^		*0.85*		*0.87*		*1.0*		*0.75*		*1.0*			*1.0*		*1.0*		*1.0*		*1.0*	
**Glaucoma (DemL)**																								
	Machine learning model and deep learning model	MC^f^		*0.99*		*0.99*		*0.98*		*0.99*		*0.99*		*0.99*			*0.99*		*0.98*		*0.99*		*0.99*	

^a^AUC: area under the curve.

^b^MC: multiclass classification.

^c^BC: binary classification.

^d^Second best score.

^e^Best scores are italicized.

^f^MC: multilabel classification.

### Comparative Analysis of Computational Time

We have performed a comparative analysis of the processing time between the LLM and human annotators, which has unveiled intriguing insights. This analysis delves into the time required for classification based on the abstract, the title, and both the title and abstract of scientific articles. Manually annotating articles is a time-consuming task, as human annotators require a significant amount of time to label each article. The overall process can span over several weeks, depending on the number of articles and the number of categories for annotations. For instance, annotating the abstract of 1 article with 2 categories may take, on average, 4 to 5 minutes. However, automated models take notably less time to complete similar tasks (Table 6).

Notably, using both title and abstract as input led to a slightly increased processing time for the BART model, although it remained significantly faster than human annotation. We conducted 2 types of trend analysis: category wise and timewise. These analyses can be applied to any classified category and can highlight different trends in a concise and quick manner. A report is generated at the end, encompassing user-specified inclusion criteria and other relevant aspects to aid researchers (Figure S3 in Multimedia Appendix 1).

**Table 6:** Comparative analysis of processing time by large language model (Bidirectional and Auto-Regressive Transformers [BART]) and human annotator.

Data set and category			Articles, n (%)		Abstract (minutes)		Title (minutes)		Title and abstract (minutes)		Annotation by human (minutes [approximately])		Timeline (months)
**Retinal disease**													
	Clinical, experimental, and automated model	1000 (100)		141.50		15.21		194.2		3000		4	
	DR^a^, DME^b^, AMD^c^, glaucoma, dry eye, cataract, CSR^d^, and retinal detachment	1000 (100)		274.06		27.30		283.43		4000		4	
	Screening, diagnosis, prognosis, etiology, and management	464 (59)		118.71		13.11		120.34		1600		4	
	Image processing techniques, machine learning model, and deep learning model	156 (15.6)		21.23		2.45		23.78		400		4	
**Dry eye**													
	Tear film break up time, infrared thermography, lipid layer interface pattern, meibomian gland study, tear film assessment, and tear meniscus assessment	67 (100)		36.45		6.45		37.12		2800		2	
**Glaucoma (DemL)**													
	Deep learning model and machine learning model	115 (100)		19.30		1.83		32.23		1000		1	

^a^DR: diabetic retinopathy.

^b^DME: diabetic macular edema.

^c^AMD: age-related macular degeneration.

^d^CSR: central serous retinopathy.

### Limitations

The limitation of this study is that we have included articles from the PubMed database, which may have resulted in the exclusion of relevant articles related to the chosen keyword. However, future plans involve the integration of additional databases such as Google Scholar, IEEE Xplore, and Springer to address this limitation and ensure a more comprehensive coverage of relevant literature. Articles from various databases can unveil trends and patterns that transcend specific domains, increasing the applicability of the findings.

We used the ZSL model for text classification. As the model is not specifically trained for downstream tasks, there are several potential biases and challenges to consider. The model may not fully comprehend the semantics or nuances of the new task, leading to biases in predictions or misinterpretations. Downstream tasks have different data distributions compared to the original ZSL task, causing the model to struggle with new patterns or biases.

We selected BART as the ZSL model, as it is the latest open-source model. BART pretraining results in the creation of semantic embeddings that capture various linguistic nuances. These embeddings enable the model to understand the semantics of different tasks, even for those tasks it has not been explicitly trained on. Its task-agnostic pretraining allows it to be adapted for various other fields by providing task-specific prompts during inference. Leveraging BART for zero-shot tasks often involves careful prompt engineering. By formulating prompts that guide the model to perform specific tasks or make predictions for unseen classes, users can harness the model’s prelearned linguistic capabilities. This can be addressed by carefully designing the prompt and adding a little description instead of using 1 word for each category, and then the model will perform better (Table 3). A limitation of the ZSL is its potential to exacerbate inequality. If the categories are not carefully designed, ZSL models may unintentionally reinforce inequalities by favoring certain classes or groups. This can also be addressed by prompt engineering. Another limitation we found is that the ZSL model will face difficulties in performing multilabel classification when categories are closely related. This issue can be resolved by dividing the multilabel classification into binary classification for each class.

The computational demands and knowledge cutoff limitations in LLM are also constraints that can be addressed by leveraging reinforcement learning techniques. The implementation of adaptive learning, active learning strategies, and exploration-exploitation balancing is being explored to address computational challenges while minimizing the impact of the knowledge cutoff. In addition, user-driven reinforcement and collaborative efforts within the research community are being incorporated to refine the models. These reinforcement learning strategies are expected to enhance the adaptability, efficiency, and overall performance of LLM, ensuring its continued relevance and effectiveness.

### Conclusions

We developed a framework based on BART ZSL for the categorization and trend analysis of articles and demonstrated a proof-of-concept scenario in the field of ophthalmology. We used the ZSL model for the categorization of articles in the field of ophthalmology, but it is extendable to other categories and fields without requiring any additional training. The model can generalize to new classes it has not observed during training, making it adaptable to different domains and applications.

The results demonstrated that the model achieved promising outcomes across most categories. In addition to article classification, trend analysis highlighted the evolution of technology in ophthalmology. Accurate and quick classification of scientific papers enables efficient information retrieval, allowing researchers to access relevant studies more quickly and obtain insights into the trend of technology and future directions. Future research directions include exploring more specialized LLMs for further improvement. In addition to this, we also have plans to develop an automated literature review tool. This tool aims to streamline and enhance the literature review process by incorporating advanced algorithms to efficiently analyze and summarize relevant research findings.

## Appendix

Multimedia Appendix 1 Supplementary dataset, tables, and figures.

## Abbreviations

AUC: area under the curve
BART: Bidirectional and Auto-Regressive Transformers
BERT: Bidirectional Encoder Representations from Transformers
DR: diabetic retinopathy
GPT: Generative Pretrained Transformer
LLM: large language model
mDeBERTa: Decoding-enhanced Bidirectional Encoder Representations from Transformers with Disentangled Attention
MeSH: Medical Subject Headings
NLP: natural language processing
RenD: retinal diseases
ZSL: zero-shot learning

## Data Availability

The data set (RenD) generated during this study is available in the Mendeley Data repository [23].
