# Supplementary material for: Automated Category and Trend Analysis of Scientific Articles on Ophthalmology Using Large Language Models: Development and Usability Study
Source: JMIR Form Res. 2024 Mar 22;8:e52462. doi: 10.2196/52462 (PMC10998173; doi:10.2196/52462)
Supplement: Multimedia Appendix 1 [file formative_v8i1e52462_app1.docx]

Contents

[Appendix A 1](#_Toc156226409)

[1.Dataset RenD 1](#_Toc156226410)

[2.Dataset RenD Annotation Guidelines 1](#_Toc156226411)

[Appendix B 3](#_Toc156226412)

[1.BART Architecture 3](#_Toc156226413)

[2. BERT and Hyperparameter Tuning 4](#_Toc156226414)

[Appendix C 4](#_Toc156226415)

[1. Results: Trend Analysis 4](#_Toc156226416)

[2. Results: Categorization of DEye Dataset 5](#_Toc156226417)

# Appendix A

This section explains about the data collection and data annotation process.

## 1.Dataset RenD

We have collected 1000 articles from PubMed. We searched the keywords diabetic retinopathy (DR), glaucoma, diabetic macular edema (DME), age-related macular degeneration (AMD), cataract, dry eye, retinal detachment, and central serous retinopathy (CSR). For each keyword we have collected 150 articles, and selected the 100 articles from each disease to prepare a balanced dataset, and the rest of 200 images were randomly selected from the all the diseases. These 1000 articles are divided into three sets and given to three teams. Each team has three graders (one ophthalmologist, two medical doctors), all of them provide annotation for each article against 28 labels (see Table A1). During the annotation process if extracted article does not fall under any of the diseases then we have removed that article from the dataset. The final label was decided based on voting, at least two of the graders should agree on the label.

## 2.Dataset RenD Annotation Guidelines

In order to maintain consistent and fair annotation, we have supplied guidelines to instruct graders in the data annotation process. The specific criteria for different categories can be found in Table A1.

Table S1. Guidelines for data annotations into different categories

| Class/Categories |  |
| --- | --- |
| Clinical  Study | A clinical study that involves human participants. |
| Experimental  study | A study that involves animals for evaluation. |
| Review study | All types of review articles.   - Systematic review. - Meta-analysis. - Literature Review. - Scoping review. - Rapid review. - Umbrella review. - Systematized Review. |
| Automated Study | Studies based on some automated model (AI/Image processing techniques). Statistical analysis-based studies are not included in this category. |
| Modality | Mention the modality of study.   1. Fundus, 2. OCT, 3. OCTA, 4. FAF, 5. VF |
| DR | Diabetic Retinopathy |
| DME | Diabetic macular edema |
| Glaucoma | Glaucoma |
| AMD | Age-related degeneration |
| Cataract | Cataract |
| CSR | Central serous retinopathy |
| Retinal Detachment | Retinal Detachment |
| Keratoconus | Keratoconus |
| Dry Eye | Dry Eye |
| Other Corneal Conditions | Other than Keratoconus and dry eye |
| Screening | The study that involves some screening methods for detecting suspect of retinal disease. |
| Etiology | A study describes the cause of a retinal diseases. |
| Genetics | Involves scientific studies individual genes or groups of genes and their effects on retinal diseases. |
| Diagnosis | A research study that evaluates methods of detecting disease. |
| Management | 1. **Prevention:** The studies present and discuss the prevention methods for retinal disorders. 2. **Drug:** Studies involves test of new drugs, already approved drugs, and devices. 3. **Therapy:** Study discussing the therapy (non-surgical treatment) for retinal diseases. 4. **Surgery:** Articles investigate different surgical procedures and their outcomes for retinal diseases. |
| Prognosis | The study discusses the likely outcome or course of a disease; the chance of recovery or recurrence. |
| Digital Image  Processing  Techniques | Study that employed only digital image processing techniques for detecting retinal lesions.  **For example:**   - Correcting Illumination - Detecting Edges - Thresholding - Wavelet transform - Mathematical Morphology etc |
| Conventional Machine learning model | Framework using the conventional machine learning model.  **For example:**   1. Linear Regression 2. Logistic Regression 3. Decision Tree 4. SVM 5. Naive Bayes 6. kNN 7. K-Means 8. Random Forest 9. Dimensionality Reduction Algorithms 10. Gradient Boosting algorithms |
| Deep learning Model | Models based on Convolutional Neural Networks (CNN), Long Short-Term Memory Networks (LSTMs), Recurrent Neural Networks (RNNs), and Generative Adversarial Networks (GANs) etc. |
| DL Architecture | Following are the DL architectures:   1. VGG Net 2. ResNet 3. U-Net 4. PSP-Net 5. Dense Net 6. Inception Net 7. Xception Net 8. Google Net |
| Supervised  Model | Models that used labeled datasets to train algorithms that to classify data or predict outcomes.  **Example of Machine learning supervised models**: decision tree, logistic regression, linear regression, support vector machine, Random forest etc |
| Unsupervised  Model | Models that used unlabeled data to discover hidden patterns or data groupings without the need for human intervention.  **Examples of unsupervised models**: dimension reduction (Principal Component Analysis (PCA)) and clustering (K-Means Clustering, Hierarchical Clustering). |
| Quantification | 1. **Segmentation** **Model (SM):** The model that extracts the different retinal pathologies but does not perform any classification of retinal disease. For example: Study proposed a segmentation model for optic cup and disc but don’t perform any classification into retinal disease. 2. **Classification** **Model (CM):** The model that performs classification of retinal disease either based on quantification or without quantification. |

# Appendix B

## 1.BART Architecture

BART adopts the conventional sequence-to-sequence Transformer framework. However, in a manner similar to GPT, it replaces ReLU activation functions with GeLU activations, as suggested. Additionally, BART initializes its parameters using a normal distribution with a mean of 0 and a standard deviation of 0.02. In the base version of the model, both the encoder and decoder consist of 6 layers, whereas in the large model variant, each has 12 layers. The architecture of BART closely resembles that of BERT, with a couple of notable distinctions: (1) In BART, each layer of the decoder also conducts cross-attention on the final hidden layer of the encoder, a feature characteristic of the transformer sequence-to-sequence model. (2) Unlike BERT, which incorporates an additional feed-forward network prior to word prediction, BART does not include this component. Overall, BART possesses approximately 10% more parameters compared to a BERT model of similar size. BART is trained through a process where documents are intentionally corrupted, followed by optimizing a reconstruction loss. This loss is measured as the cross-entropy between the output of the decoder and the original, uncorrupted document. Differing from existing denoising autoencoders that are designed for specific types of noise, BART provides the flexibility to employ any form of document corruption. In extreme scenarios where all information about the source document is obliterated, BART effectively functions as a language model.


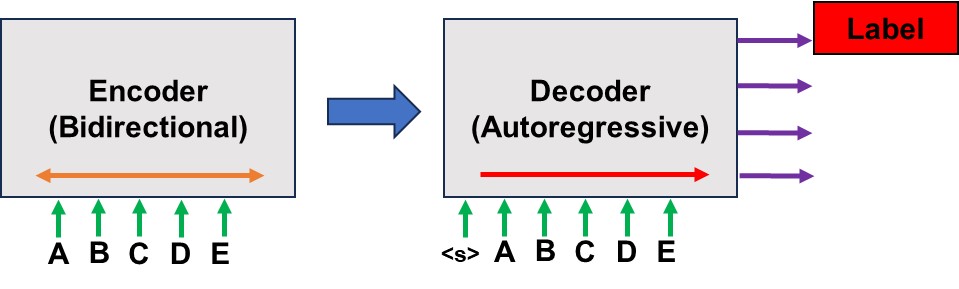


Figure S1. Illustration of BART Architecture that is used for the classification tasks, same input is fed into the both encoder and decoder, and the first bit from the decoder is considered as output label.

## 2. BERT and Hyperparameter Tuning

For fine tuning the BERT model, we have divided the data into train (80%) and test data (20%). Within train data, 70% of data is used for training the model and 30% for validation.

**Table S2: BERT model selection and hyperparameter tuning.**

| Model | Epochs | Learning rate | Batch size | Max_length | threshold | AUC | FI micro  score |
| --- | --- | --- | --- | --- | --- | --- | --- |
| Base | 17 | 1e-06 | 12 | 512 | 0.6 | 0.70 | 0.55 |
| DistillBERT | 25 | 1e-06 | 12 | 512 | 0.6 | 0.5 | 0.38 |
| SciBERT | 20 | 1e-05 | 8 | 400 | 0.55 | 0.71 | 0.57 |
| Pubmed  BERT | 20 | 1e-05 | 8 | 400 | 0.59 | 0.68 | 0.52 |
| BioBERT | **20** | **1e-05** | **8** | **400** | **0.6** | **0.79** | **0.63** |

# Appendix C

1. Results: Trend Analysis We can perform several trend analysis on all the categories, Fig .C1 shows the number of articles for different ocular diseases between the time period of 2020 and 2022. It shows the more studies are published related to cataract and followed by DR and dry eye.


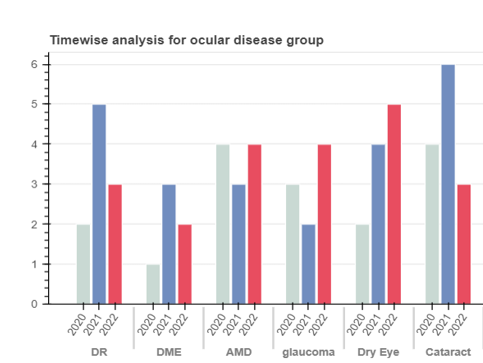


Fig S2. Showing the trend analysis of classified ocular diseases, DR, DME, AMD, glaucoma, dry eye and cataract over the period of 2020 to 2022.

## 2. Results: Categorization of DEye Dataset

The multilabel classification is tackled as binary classification. We have tested the model on various threshold values, Table 2 shows the results of BART model on various threshold values for classification of article based on abstract and title.

**Table S3: Classification results at various threshold values for all categories for DEye dataset.**

| Dataset | Categories | th | Abstract | | | | | Title | | | | |
| --- | --- | --- | --- | --- | --- | --- | --- | --- | --- | --- | --- | --- |
|  |  |  | Ac | F1 | AUC | Pv | Re | Ac | F1 | AUC | Pv | Re |
| DEye | Tear Film Break Up Time | 0.55/0.015 | 0.83 | 0.56 | 0.76 | 0.53 | 0.58 | 0.59 | 0.44 | 0.72 | 0.29 | 0.91 |
|  |  | 0.6/0.02 | 0.85 | 0.58 | 0.74 | 0.58 | 0.58 | 0.67 | 0.50 | 0.76 | 0.34 | 0.91 |
|  |  | **0.7/0.05** | **0.912** | **0.73** | **0.79** | **1.0** | **0.58** | **0.70** | **0.47** | **0.72** | **0.34** | **0.75** |
|  | Infrared Thermography | **0.55/0.2** | **0.94** | **0.77** | **0.91** | **0.7** | **0.87** | **0.83** | **0.54** | **0.68** | **1.0** | **0.37** |
|  |  | 0.6/0.015 | 0.92 | 0.70 | 0.84 | 0.66 | 0.75 | 0.83 | 0.47 | 0.74 | 0.38 | 0.62 |
|  |  | 0.85/0.1 | 0.94 | 0.75 | 0.85 | 0.75 | 0.75 | 0.83 | 0.42 | 0.69 | 0.39 | 0.5 |
|  | Lipid Layer Interface Pattern | 0.5/0.05 | 0.79 | 0.46 | 0.73 | 0.35 | 0.66 | 0.77 | 0.34 | 0.63 | 0.28 | 0.44 |
|  |  | 0.55/0.15 | 0.85 | 0.54 | 0.77 | 0.46 | 0.66 | 0.85 | 0.28 | 0.58 | 0.4 | 0.22 |
|  |  | **0.7/0.1** | **0.91** | **0.57** | **0.71** | **0.8** | **0.44** | **0.86** | **0.47** | **0.68** | **0.5** | **0.44** |
|  | Meibomian Gland Study | **0.5/0.05** | **0.92** | **0.87** | **0.90** | **0.89** | **0.85** | **0.92** | **0.87** | **0.90** | **0.89** | **0.85** |
|  |  | 0.55/0.055 | 0.89 | 0.81 | 0.85 | 0.88 | 0.75 | 0.92 | 0.87 | 0.90 | 0.89 | 0.85 |
|  |  | 0.6/0.6 | 0.89 | 0.81 | 0.85 | 0.88 | 0.75 | 0.92 | 0.87 | 0.90 | 0.89 | 0.85 |
|  | Tear Film Assessment | 0.5/0.4 | 0.73 | 0.57 | 0.75 | 0.44 | 0.80 | 0.71 | 0.42 | 0.62 | 0.38 | 0.46 |
|  |  | **0.55/0.6** | **0.80** | **0.64** | **0.80** | **0.54** | **0.80** | **0.71** | **0.42** | **0.62** | **0.38** | **0.46** |
|  |  | 0.6/0.7 | 0.80 | 0.62 | 0.78 | 0.55 | 0.73 | 0.74 | 0.41 | 0.62 | 0.42 | 0.4 |
|  | Tear Meniscus Assessment | 0.5/0.4 | 0.98 | 0.88 | 0.99 | 0.80 | 1.0 | 0.98 | 0.88 | 0.99 | 0.8 | 1.0 |
|  |  | **0.55/0.55** | **0.98** | **0.85** | **0.87** | **1.0** | **0.75** | **1.0** | **1.0** | **1.0** | **1.0** | **1.0** |
|  |  | 0.6/0.6 | 0.97 | 0.66 | 0.75 | 1.0 | 0.5 | 1.0 | 1.0 | 1.0 | 1.0 | 1.0 |

1. Report Generation

Through the generation of a comprehensive PDF report, we have streamlined the literature review process, offering researchers and clinicians a rapid and easily digestible perspective on diverse findings. This automated approach not only enhances efficiency but also facilitates a clearer understanding of research and clinical insights, contributing to informed decision-making and knowledge dissemination.


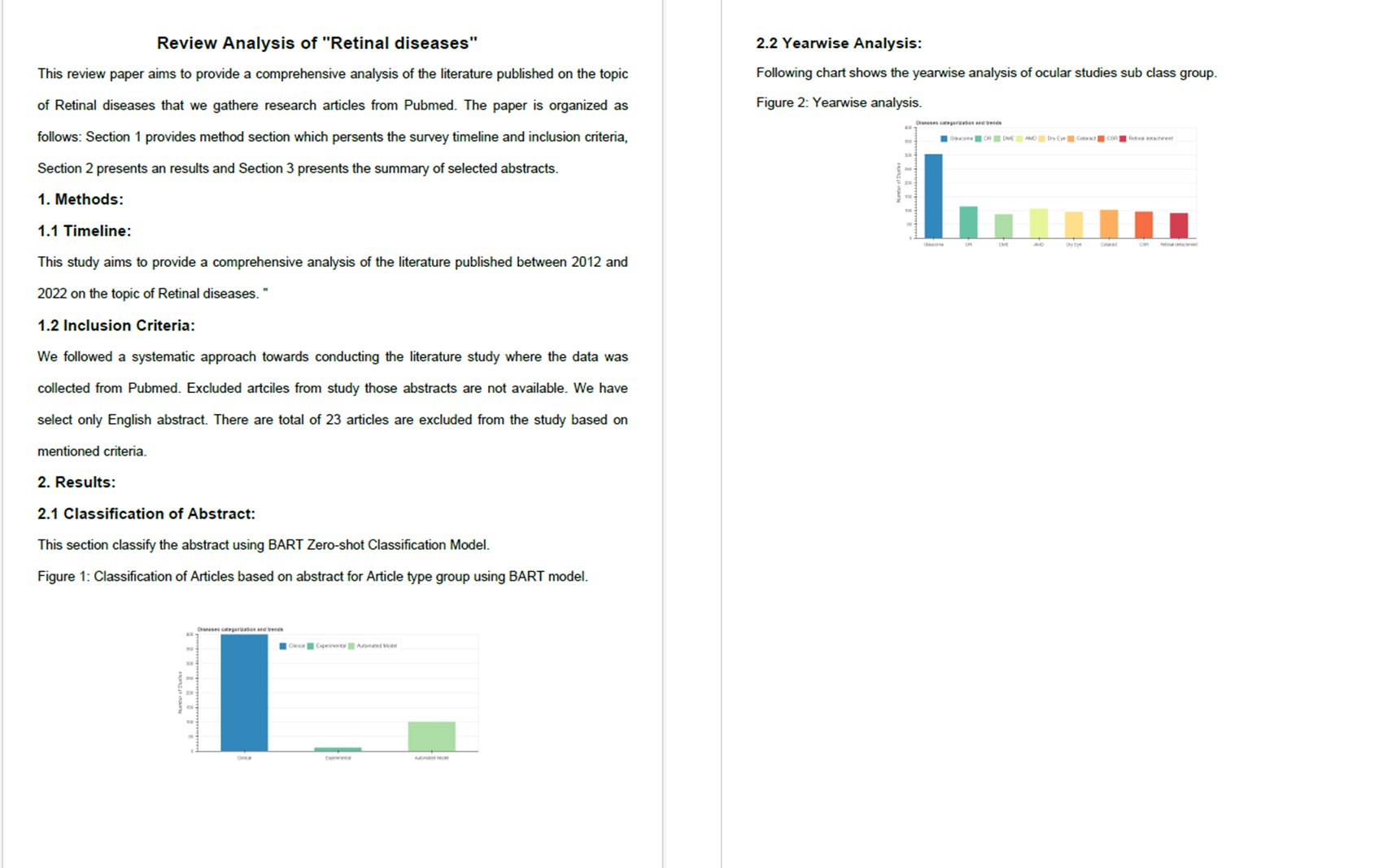


Figure S3. Generated report for quicker and easily understanding for researcher
